# Factors influencing the perceived importance of oral health within a rural Aboriginal and Torres Strait Islander community in Australia

**DOI:** 10.1186/s12889-020-08673-x

**Published:** 2020-04-17

**Authors:** Anna Tynan, David Walker, Taygan Tucker, Barry Fisher, Tarita Fisher

**Affiliations:** 1 Research Fellow, Research Support Team, Baillie Henderson Hospital, Darling Downs Health, Corner of Torr and Hogg Street, Toowoomba, Queensland Australia; 2 Honorary Research Fellow, The Rural Clinical School, The University of Queensland, West Street, Toowoomba, Queensland Australia; 3 Honorary Research Fellow. School of Dentistry, Faculty of Medicine and Health, University of Sydney, Camperdown, New South Wales Australia; 4 Dental Therapist, Oral Health Services – Nhulunbuy, Top End Health Services, Northern Territory Government Endeavour Square, Gove Dental Clinic, Endeavour Square, Nhulunbuy, Northern Territory Australia; 5 Indigenous Liaison Officer, Kingaroy Hospital, Darling Downs Health, 166 Youngman Street, Kingaroy, Queensland Australia; 6 Health Service Manager, Darling Downs Health, PO Box 361, Murgon, Queensland Australia

**Keywords:** Australia, Oral health, Indigenous, Health service utilisation

## Abstract

**Background:**

Indigenous Australians suffer from higher rates of oral disease and have more untreated dental problems and tooth extractions than the general population. Indigenous Australians also have lower rates of accessing oral health services and are more likely to visit for a problem rather than a check-up. Multiple issues effect health service and prevention programs including: characteristics of health services such as distances to health services; existence of social and cultural barriers; available wealth and social support; and, characteristics of the individual and community including the importance given to the disease. This paper seeks to explore the perceived importance of oral health within a rural Indigenous community in Australia and the factors influencing this perception.

**Methods:**

The study used a phenomenology research design incorporating focus group discussions and in-depth interviews. It was undertaken in partnership with communities’ Health Action Group who guided the focus, implementation and reporting of the research. A convenience sample was recruited from established community groups. Thematic analysis on the transcripts was completed.

**Results:**

Twenty-seven community members participated in three focus groups and twelve in-depth interviews. The study found that the community gives high priority to oral health. Factors influencing the importance include: the perceived severity of symptoms of oral disease such as pain experienced due to tooth ache; lack of enabling resources such as access to finance and transport; the social impact of oral disease on individuals including impact on their personal appearance and self-esteem; and health beliefs including oral health awareness. Participants also noted that the importance given to oral health within the community competed with the occurrence of multiple health concerns and family responsibilities.

**Conclusion:**

This paper highlights the high importance this rural Indigenous community gives to oral health. Its findings suggest that under-utilisation of oral health services is influenced by both major barriers faced in accessing oral health services; and the number and severity of competing health and social concerns within the community. The study results confirm the importance of establishing affordable, culturally appropriate, community-based oral health care services to improve the oral health of rural Indigenous communities.

## Background

In Australia, Aboriginal and Torres Strait Islander peoples (respectfully referred hereafter as Indigenous) suffer from higher rates of oral disease including dental caries and periodontal disease than the non- Indigenous population [1–4]. Oral disease causes pain and disfigurement, has a negative influence on quality of life, and is linked to poor nutrition, diabetes, and cardiovascular disease [5, 6]. In addition to this, Indigenous Australians are known to have a higher disease burden than non-Indigenous. This is complicated further by a higher prevalence of social concerns including higher level of psychosocial risk factors; lower socioeconomic status; lower social capital; and a higher prevalence of lifestyle risk factors [7, 8].

Indigenous Australians have lower rates of dental visits and are more likely to visit for a problem than for a check-up than the general population [9, 10]. Low levels of individual preventive oral health care and engagement in high risk lifestyle activities has also been reported among Indigenous Australians [11, 12]. This includes high levels of smoking, alcohol consumption, diets high in sugar, and poor reported engagement in oral hygiene activities [11, 13–15]. Research indicates that broader structural and social factors including social capital inform oral health choices and impact on poor uptake of preventative oral health measures [14, 16]. Having a healthy diet and access to oral hygiene equipment are influenced by living arrangements and limited budgets within Indigenous communities [17, 18]. Evidence exists of insufficient education about oral disease prevention due in part to difficulties accessing oral health services, with research also indicating that some Indigenous Australians believe having poor oral health was normal [9, 10, 17].

Multiple issues affect individual and community uptake of health services and disease prevention activities including factors associated with the disease [19–23]. Factors associated with the disease include the severity of a disease’s impact on individuals and communities; and its prevalence [22]. Individual and community factors influencing treatment seeking behaviour include the perceived importance of the disease and access to financial resources [22, 24]. Anderson and Davidson (2014) identify multiple factors influencing perceived importance of a disease including social characteristics such as ethnicity, education or historical context; access to enabling resources such as finance and transport; health beliefs including health attitudes and knowledge; and, the perceived severity of symptoms of the disease [22]. Health service factors have also been shown to influence the decision to access oral health services within Indigenous communities. These factors include high costs associated with accessing treatment; lack of familiarity with the dental care provider; and lack of availability of culturally appropriate services [25–27].

Factors associated with the broader socio-economic and historical environment include employment status, income, education, self-efficacy, health literacy and cultural connection which all have a strong influence on both the patterns of oral disease for Indigenous people in Australia [28–32]; and on the uptake of oral health services [33–37]. Importantly, historical and ongoing conflict over landownership associated with colonisation also play a major role in disparities in oral health outcomes and oral health service uptake [38–40]. This has been highlighted by The Australian Research Centre for Population Oral Health which has stated that, ‘Aboriginal and Torres Strait Islander peoples present with third world problems in a first world country as a result of the dispossession of their land, disruption of their culture, material deprivation and racial discrimination’ [41].

Rural and remote Indigenous communities are faced with further challenges due to the limited availability of oral health services leading to greater distances to travel, and the limited consistency of rural and remote oral health services due to workforce shortages, staff turnover and already existing heavy workload which in turn influences relationship and partnership building with the community [42, 43]. Most dental health preventive services require professionally trained personnel, needing equipment and regular availability of the service for the re-application of treatment [44]. In disadvantaged and especially remote communities with limited access to services and staff, this is usually not possible. Further to this, rural Indigenous communities face a significant number of other competing health priorities associated with major diseases including cardiovascular disease, diabetes, kidney disease, respiratory disease, cancer and mental illness [45].

The purpose of this paper is to explore the perceived importance of oral health within a rural Indigenous community and the factors influencing this perception. This is considered in the context of the barriers to accessing oral health care and prevention, and the multiple competing priorities faced within rural Indigenous communities. This study is one aspect of a larger qualitative study exploring oral health service needs; the community’s perceptions of the importance of oral health; and, how oral health can be best improved within a rural Indigenous population.

## Methods

### Study design

A phenomenological research design was employed to guide the conduct of the study. Phenomenology aims to understand the essence of the lived experiences of persons about a phenomena [46]. In-depth interviews (IDIs) and focus group discussions (FGDs) were undertaken to explore the perceived importance community members gave to oral health and its influencing factors. These methods have been well documented as appropriate for focusing on topics that are not well explored and working with populations on topics that require rich contextual understandings [47]. The following section is described based on the consolidated criteria for reporting qualitative research (COREQ) [48].

### Setting

This study was conducted in a small, rural Indigenous community in Queensland, Australia. The community has a population of approximately 1300 people of which over 95% identify as Aboriginal and 2.5% identifying as both Aboriginal and Torres Strait Islander. Because of the relocation of Indigenous people under past government policies the population have connections to many different Australian Indigenous nations. Just under a quarter of the community earn an income other than government benefit and around 15% of residents have completed year 12 or equivalent [49].

Within the community, the local Aboriginal Controlled Community Health Service provides oral health services intermittently throughout the year mainly focused on treatment and, there is a visiting state government funded school dental van providing oral health check-ups and treatment to children. The Royal Flying Doctors also provide an annual 10 day visit for check-up and treatments which commenced around the time of the study. The closest private dental services are located approximately 8 km from the community and the closest public dental services are located approximately 50 km from the community.

### Research team

This study was undertaken by a research team including Indigenous and non-Indigenous persons in partnership with the local community’s Health Action Group. The Health Action Group includes representatives from the community (e.g. Elders and parents) and from the diverse agencies including health, education and welfare services. The Indigenous research team members were also members of the local community with extensive experience in health service provision. Their participation supported all stages of research development, implementation and reporting. This included a key role in raising awareness of the project to facilitate participant recruitment and in assisting with recruitment of a local research assistant.

### Study participants and recruitment

Following advice from the local Health Action Group and key informants within the local community, a convenience sample was recruited from established groups. Differing community groups were strategically included to capture the diversity and breadth of oral health experience within the community. These groups included participants accessing chronic disease services, attending hospital outpatient’s services, maternal health services and other community groups. A local Indigenous research assistant from the community was employed to assist with managing recruitment and supporting the discussion moderator to conduct individual in-depth interviews and focus group discussions including taking observational notes.

Many potential participants initially heard about the study though advertising via their respective community groups. Interested participants contacted the local research assistant who provided them with additional verbal and written information face to face. To allow for the possibility that participants may not be comfortable talking openly in a group about the topic, interested participants were provided with the choice of participating in focus groups or individual discussions. Informed consent was completed at the time of research discussion. This process was guided by the discussion moderator who was a member of the research team with assistance from Indigenous team members and the research assistant.

### Data collection

Where possible, focus groups were organised to achieve relative homogeneity regarding age, engagement with specific health service, gender or role within community. This was to further facilitate participant comfort in the group environment and maximise topic understanding [50]. Data collection through these two types of qualitative methods was completed from January – April 2017. The discussion moderator was a member of the research team and had an established relationship with the community at the time due to her role as a dental therapist within the community. The participants were recruited from groups unrelated to her professional role. The discussion moderator was supervised and trained by two other members of the research team who were both experienced qualitative researchers. This included recording practice interviews and reviewing them as a team to discuss areas for improvement to ensure effectiveness of technique. Focus groups were held at community venues where participants were already attending including facility meeting rooms. Individual interviews were held at local venues suggested by participants and included community meeting rooms. Only the participants and the researchers were present during the discussions. Discussions lasted 30–45 min.

The schedule of research questions to guide both individual interviews and focus groups was developed in partnership with the local Health Action Group. The development of questions was based on a review of the literature, discussions within the research team and with the Health Action Group and, informal discussions with local community members to ensure cultural appropriateness and clarity of questions. For this component of the research project, questions centred around two foci, the perceived importance of oral health and the factors influencing the importance given to oral health (Additional file 1). The questions were piloted by the discussion moderator with team members from the local community.

Discussions were facilitated to allow for a relaxed conversational process that aimed to build a relationship with the participants. Participants were encouraged to share their personal perceptions and stories in order to co-develop knowledge and share experiences simulating a yarning or storytelling experience in the context of Australian Aboriginal and Torres Strait Islander people [51, 52]. The interview questions schedule was refined during the study to allow for reflexivity of the research process [53]. Field notes were completed after each research activity by the researchers present and throughout the entire research process to assist with recording investigator observations and reflections. Data analysis occurred alongside the data collection period and data saturation was determined when no new themes emerged from the data.

### Data analysis

All in-depth interviews and focus groups were transcribed verbatim. Thematic analysis applying Braun and Clark’s six step process was performed to make essential meanings of the phenomena which included familiarisation with the data; succinct coding of the data; searching for themes; reviewing of themes; defining and naming of themes; and writing up findings [54]. Data analysis initially involved two of the research team members familiarising themselves with the data by reading the transcriptions independently to gain an understanding of participants’ perceptions and experiences in order to get a global sense of the data. The transcripts were then read a second time to gain a greater understanding of participants experiences and, extract codes from each transcript. The underlying meaning of each code was formulated using inductive reasoning where meanings were generated through observations of patterns and relationships. The formulated meanings were then organised in clusters of themes. The two researchers compared their proposed codes, discussed similarities and differences, and checked them against original transcripts for validation until consensus was reached on emerging themes. The proposed themes were then reviewed and confirmed by a member of the research team who was also a community member to support accuracy of interpretation and to further explore interpretation where necessary. The entire process involved total emersion in the data for as long as needed in order to ensure a pure and thorough description of the phenomena. Organisation of the data was completed with assistance of NVivo© 12 (Windows) QRS and written up as findings. Feedback on the draft findings was sought from the local Health Action Group and adjustments made as indicated. Analysis and description within each theme were continued until consensus was reached by the entire research team.

### Research ethics

This research was conducted within the guidelines for Ethical Research in Australian Indigenous Studies [55] and was granted ethics approval from the Darling Downs Health Human Research Ethics Committee (Protocol HREC/16/QTDD/42). The study was instigated following an expressed interest by the community through the Health Action Group. The research team included members of the local rural Indigenous community. These members and the local Health Action Group were integral to providing advice in relation to the study including: study design; development of the data collection instruments; promotion of the study in the community; assistance with cultural support of participants if needed and responding to any concerns if they arose; and reporting to the community. Prior to conducting any session, the project and implications of involvement were reviewed with potential participants. Written informed consent was obtained from all participants. All data were de-identified, and the reporting of the findings was submitted to the local Health Action Group for discussion.

## Results

Three focus groups and twelve in-depth interviews were completed with twenty-seven community members. There was a total of 10 male and 17 female participants. Four participants were aged between 18 and 39 years, while the remaining 23 were aged over 40 years.

Nearly all of the respondents gave a high level of importance to oral health.

*Yes, it is, very important … because it’s like a part of you.*

IDI 3 Female Participant

Four key themes were identified as influencing the importance given to oral health. These included: the *severity of symptoms* of oral disease, in particular pain; the *demand on resources to respond to oral disease* such as financial resources; the severity of the *social impact* of oral disease such as the damaging effect on personal appearance; and individual’s *health beliefs and oral health awareness*.

### Severity of symptoms

Participants gave a high importance to oral health principally because of the pain associated with untreated oral disease. These perceptions stemmed from participants’ personal experience of oral disease or their observations of the impact oral disease had on their family or other community members. We suggest it would be difficult to overestimate the experience of the pain of untreated oral disease within the community as one participant described:

*You know, it’s a pain you can’t handle … tooth ache … Everybody knows how it is powerful. You lay in bed and you’re in frigging agony in your mouth. … That’s what it is, hey … Yeah, it was a pain I’ll never ever forget.*

FGD 2 Male Participant.

The severity of the ongoing pain of untreated oral disease led some participants drinking alcohol to reduce the pain of pulling out their own teeth with pliers.

*… that night, you know my tooth was aching, yeah, so what I do yeah, I just get drunk and just pull it out … then I just get the pliers and just yanked on it … that’s three times I did that.*

FGD 2 Male Participant

Participants were very aware of the suffering of other family and community members, with oral disease described as occurring frequently and often ongoing

*It’s something that I see the kids suffering with, and I know that there’s nowhere for them to go. … I actually had boys sitting on the carpet about a week and a half ago comparing abscesses and gum boils. “I have a lump here.” “Oh, well look at mine.” … It’s not nice to see the kids dealing with that sort of stuff.*

IDI 2 Female Participant

*… with the amount of holes in teeth that the kids are showing me now, and the number of abscesses and stuff, I can definitely see at some point these kids are going to be missing out on school because of their pain and their dental issues.*

IDI 3 Female Participant

### Demand on resources to respond to Oral disease

Responding to oral disease places demands on individual family and community resources including financial resources, social support and networks, transportation and the time needed to access services. The widespread experience of financial burden associated with oral disease was a principal influence on the high importance given to oral health.

*Yeah (having good teeth) is very important because when I had to pay for dental things, it cost almost 1000 bucks … It is very expensive.*

FGD 2 Male Participant.

Responding to oral disease was also recognised as placing a burden on the whole family. As one participant explained this burden includes relying on others for transport to and from an oral health service and taking up their time which can lead to lost work hours.

*If they’re not mobile then how the hell are they going to get here? So they’re just relying on family and stuff. Then family have to stay there with them, which is a concern because they’re doing their own thing.*

IDI 7 Female Participant.

### Social impact

The importance of oral health was also linked to social impact most notably the benefits to appearance and self-esteem of a healthy mouth.

… *It makes you look good.*

IDI 3 Female Participant.

Oral disease was perceived to have a severe negative impact on appearance through the presence of severely stained, broken or missing teeth.

*… everybody cares about how they look. Like even old people do. Yeah. It is important to them and it’s definitely for comfort because you’ve got to eat every day. You want your teeth to eat every day. It’s about how you see yourself. It’s okay if people - how they see you, it’s all right and you can take that how you take it. But it’s important from how you see yourself and how [you like yourself]. Anything that improves especially eating and how you look. It’s an everyday thing so - it has a big impact on you really -to having a better life and a better outlook on life*.

IDI 10 Male Participant.

One participant noted the importance of good oral health to her confidence.

*Yeah, that actually boosts my confidence a lot. Because I never used to smile because I had like a hole in my teeth, but ever since it’s fixed, I can smile freely.*

IDI 1 Female Participant

Oral health was also given high importance because of its importance for eating and nutrition.

*Well, yeah very important just to eat.*

*Well you need your teeth to chew you know …*

FGD 2 Male Participant

Participants also highlighted the important role of oral health care with one participant reflecting on recent care:

*Feels great, it’s like a second chance*

IDI 1 Male Participant*.*

One participant gave high importance to oral health due to her perception of the severely negative impact oral disease has on those with chronic disease in the community.

*It means a lot because we have a lot of people here in this community that have ongoing issues with diabetes and heart problems.*

FGD 3 Female Participant

### Health beliefs and Oral health awareness

Health beliefs are attitudes, values and knowledge people have about health and health services that can influence their subsequent perception of the importance of a particular disease. Knowledge about oral disease and its impact and, prevention and treatment options were observed by participants as influencing the importance given to oral health. Several participants believed that a limited number in the community did not prioritise oral health as much as they should which they associated with a lack of awareness.

*I don’t think they have a priority for oral health. … . I think that’s entrenched. It’s where we’ve got to go with education and just improve that whole understanding. I mean I’ve done some women’s health days where I’ve spoken, and these are all over the place, and it’s astounding how many people don’t realise the potential for the chronic disease impact with dental health, and stuff.*

FGD 1 Female Participant

For the older participants, reflection on their increased awareness of oral health and oral disease prevention strategies highlighted for them both the importance of oral health and missed opportunities to protect their own oral health.

*Oral health is important to me now, now that I know what I know. …*. *if you look after your teeth, you’ve got them for the rest of your life.*

IDI 5 Female Participant.

*I only wish I knew then what I know now about it. That’s why, right at the beginning, that’s what we needed to know. Otherwise we’d have looked after our teeth; ate the right food and that … So, oral health is important to me now, now that I know what I know.*

IDI 7 Female Participant.

For some older participants, new knowledge seemed to not influence their individual behaviour with many reporting they had bad teeth and yet not prioritising oral health for themselves. However, they were concerned about the next generation and wanted to ensure that younger people were aware of how to prevent oral disease, and were also able to engage in good oral health behaviours which would lead to better outcomes for them.

*My teeth are rotten, but it is (still) important. For my kids I think I push it more on them because mine are like (this) - I’m old and [it’s going to be] too late.*

IDI 8 Female Participant.

Despite the high importance given to oral health participants also noted that oral health is just one of many competing health and social issues within the community. This is clearly highlighted in a participant’s reflection about how the importance of oral health had changed for her over time.

*You only realise these things when you grow older. Because to me at the time, it wasn’t important when I was having all my children. It wasn’t important. Like I said, the only important thing was putting food on the table … Keeping them fed, and clothed, and clean.*

IDI 7 Female Participant.

## Discussion

This research explored the perceived importance of oral health within a rural Indigenous community and the factors influencing this perception. The study found that most community members give a very high importance to oral health. This high importance given to oral health is consistent with previous studies that show Indigenous communities are concerned about oral health [17]. The key factors leading to the high importance given to oral health are: the widespread experience of the severe pain of untreated oral disease; and the high cost and prevailing difficulty - and at times inability - to access oral health care. Additional factors include the experience of the positive benefits of good oral health on daily living including improved appearance, self-esteem and eating. Participants also noted that the importance given to oral health within the community varied both with the knowledge of the impact of oral health and oral disease and, with the occurrence of multiple competing health concerns and family responsibilities.

Pain was highlighted as a factor in influencing importance and stemmed from experience of episodes of severe tooth or mouth pain and discomfort. This finding is reflected in the literature with Indigenous people more likely to have visited an oral health service due to relief of pain [27, 56]. Concerns about oral disease and impact on personal presentation was also a factor influencing increased importance, a finding similar to a previous study [27]. These findings reaffirms that the mouth should not be viewed separately from the rest of the body because oral health affects general health through considerable pain and suffering and impacting on quality of life and well-being. This study also showed that although pain and the social impacts of oral disease influenced perceived importance, it did not always instigate treatment seeking at a health facility.

Nearly all participants had either experienced the negative impact of oral disease or knew of family or community members who had faced the impact of untreated oral disease. However, the literature also reports that Indigenous people are less likely than the general population to have visited an oral health service in the past year [27, 56]. This is a major concern as healthy preventive behaviours for oral disease include the regular attendance of oral health services including regular attendance for check-ups rather than dental problem. Such visiting patterns provide the opportunity for early diagnosis, prompt treatment of dental disease and provision of preventive services. This research highlights that the under-utilisation of oral health services is associated not with any limited importance given to oral health within the community, but rather with the major barriers faced in accessing oral health services and engaging in oral care prevention activities; and, the number and severity of competing health and social concerns within the community. In fact, some of the barriers such as cost and access, actually increased perceived priority for oral health.

The study results confirm the importance of establishing affordable, culturally appropriate, community-based oral health care services and oral health promotion which allow access to these health services in order to improve the oral health of rural Indigenous communities. Recognising the limitations of health promotion and prevention programs being focused at the health service level is important for these rural and remote communities. There is a need for oral health messaging and management to adapt to the challenges of maintaining good oral health when services are not available. Providing a sector wide approach to oral health including increasing opportunities for informal discussion of oral health through schools and sporting activities is important for management and sustainability in rural and remote Indigenous communities. Further research is however needed on community perceptions on how to build a sustainable community wide oral health program.

Older participants in this study reported improvements in knowledge of oral health care and prevention over time which resulted in more awareness of missed opportunities. However there was also evidence that these participants considered it was too late to do anything to improve their oral health. A previous study has reported that Indigenous people perceive dental problems as inevitable including a perception that most children will get problems with their teeth that will likely lead to extraction [17]. The suffering of children was also of great concern in the present study, with participants indicating a sense of inevitability about oral disease in children. The perceived lack of interest observed of some of the other members of the community in our study may be reflective of this belief of inevitability and should be considered in oral health promotion and care. There is a need for further research of beliefs regarding the inevitability of oral disease and its impact on accessing oral health services.

### Limitations

Representation of participants may be biased to those who have self-selected as they are concerned about oral health. Participants also tended to be older adults and so it is noted that most participants had experienced complications of oral disease. Difficulties are therefore noted in interpreting these findings and their application to youth and younger adults. The discussion moderator was a known dental health therapist in the community. Participants may have been more inclined to express their concerns due to the facilitators known role. However, the community rapport already established with the facilitator was also seen as an important component to engaging in rich discussions about the topic.

## Conclusion

The study highlights the high priority given to oral health within a rural Indigenous community. The high priority is primarily attributed to the widespread experience of the severe pain of untreated disease and the cost and difficulty of gaining treatment for oral disease. Indigenous communities have both a high burden of oral disease and reduced uptake of oral health services and engagement in preventative strategies. This study has found that a limited uptake of oral health services in this community is not due to a lack of importance given to oral health but rather to barriers faced in accessing oral health care. This finding suggests that public health services will need to consult with individual rural Indigenous communities to identify local barriers to accessing oral health services and how these can be best ameliorated. The study contributes to the body of research that recognises that the priority given to a disease within communities may be influenced by factors other than simply the pathology of the disease. It also highlights that there is a need to understand the importance given to a disease within a community and the factors influencing this in order to better serve the needs of local communities.

## Supplementary information

**Additional file 1.**

## Data Availability

The data generated during the current study are not publicly available due to the potential for individual privacy being compromised but are available from the corresponding author on reasonable request.

## Abbreviations

FGD: Focus Group Discussion
IDI: In-depth Interview
