# Supplementary material for: Factors influencing the perceived importance of oral health within a rural Aboriginal and Torres Strait Islander community in Australia
Source: BMC Public Health. 2020 Apr 17;20:514. doi: 10.1186/s12889-020-08673-x (PMC7164228; doi:10.1186/s12889-020-08673-x)
Supplement: Supplementary file 1 — Additional file 1. [file 12889_2020_8673_MOESM1_ESM.docx]

**Interview Guide for Focus group discussions and In-depth interviews with community members.**

The theme list did not rigidly structure the discussions but instead were used as a guide to the discussion where necessary.

**THE IMPORTANCE OF ORAL HEALTH**

1. **The importance of oral health to the community**

- Describe the experience of having good teeth (oral health)?
- How important is looking after your teeth to you? Your family? Your community? Why?
- What is the importance of good teeth (oral health)?
- What impact has having bad teeth had? (What has been the impact of oral disease?)

To you? Your family? Your community?

**2. Importance of oral health compared to other community issues**

How important is having good teeth (oral health) compared to other concerns – for you? Your family? Your community?

1. **Breadth and severity of impact**

- How does having good or bad teeth (oral health) impact on you? How severe is this impact?
- How does having good or bad teeth impact on your family? How severe is this impact?
- How does having good or bad teeth impact on your community? How severe is this impact?
